# Valorization of Pineapple (*Ananas comosus*) By-Products in Milk Coffee Beverage: Influence on Bioaccessibility of Phenolic Compounds

**DOI:** 10.1007/s11130-024-01183-w

**Published:** 2024-05-02

**Authors:** Zeynep Buse Kocakaplan, Gulay Ozkan, Senem Kamiloglu, Esra Capanoglu

**Affiliations:** 1 Department of Food Engineering, Faculty of Chemical and Metallurgical Engineering, Istanbul Technical University, Maslak, Istanbul, 34469 Türkiye; 2 Department of Food Engineering, Faculty of Agriculture, Bursa Uludag University, Gorukle, Bursa, 16059 Türkiye; 3 Science and Technology Application and Research Center (BITUAM), Bursa Uludag University, Gorukle, Bursa, 16059 Türkiye

**Keywords:** Pineapple waste, Bromelain, *In vitro* digestion, Food matrix, Caffeoylquinic acids

## Abstract

The industrial processing of pineapples generates a substantial quantity of by-products, including shell, crown, and core. Bromelain, a proteolytic enzyme found naturally in pineapple, including its by-products, may positively influence the bioaccessibility of phenolics from milk coffee. Therefore, this study aimed to assess how the inclusion of extracts from pineapple by-products, namely shell, crown and core, could impact the bioaccessibility of coffee phenolics when combined with milk. After measuring the proteolytic activity of pineapple by-products, the standardized in vitro digestion model of INFOGEST was employed to evaluate changes in total phenolic content, total antioxidant capacity, and individual phenolic compounds in different coffee formulations. The results showed that incorporating extracts from the crown or core in both black and milk coffee increased the bioaccessibility of total phenolics (from 93 to 114% to 105–129%) and antioxidants (from 54 to 56% to 84–87%), while this effect was not observed for the shell. Moreover, adding core extracts also enhanced the bioaccessibility of caffeoylquinic acids and gallic acid in milk coffee (from 0.72 to 0.85% and 109–155%, respectively). Overall, the findings of this study highlight that bromelain from pineapple core may have a favorable effect on the recovery of phenolic compounds in milk coffee, possibly due to its ability to cleave proteins. These outcomes point out that industrial by-products can be transformed into economic value by being reintroduced into the production process through suitable treatment instead of disposal.

**Supplementary Information:**

The online version contains supplementary material available at 10.1007/s11130-024-01183-w.

## Introduction

Pineapple (*Ananas comosus*), belonging to the Bromeliaceae family, is globally the third most consumed fruit following banana and citrus fruits [1]. Approximately 29 million tons of pineapples are produced annually, with major contributors being Indonesia, Philippines, Costa Rica, Brazil, and China [2]. Fresh pineapples have limited consumption, as 97% of the global pineapple production is utilized by the pineapple processing industry. In particular, the canning industry plays a crucial role in managing the extensive pineapple production on a global scale [3]. The industrial processing of pineapples generates a substantial quantity of by-products including shell, crown, and core [4]. By-products resulting from fruit processing compose over 50% of the total weight of the pineapple and are distributed as follows: 29–40% in the shell, 9–10% in the core, and 2–4% in the crown. While these by-products are primarily utilized in animal feed and the pharmaceutical industry, they have the potential to be transformed into value-added products. Bromelain, a proteolytic enzyme found naturally in various parts of pineapples including the shell, crown and core offers beneficial effects for both digestive and cardiovascular health [5].

Coffee (*Coffea arabica* L.), globally one of the most popular beverages, may be consumed either plain or with addition of milk. However, there is a debate on the effect of milk addition to coffee concerning the functional properties of the phenolic compounds. Majority of the studies have reported inhibitory effects of milk on phenolic compounds due to the potential interactions between milk proteins and coffee phenolics [6, 7]. Considering the fact that bromelain contains protease inhibitors [8], we hypothesized that incorporating extracts containing bromelain-polyphenol complex from pineapple by-products into milk coffee might have a favorable effect on recovery of phenolics besides contributing to waste valorization. In addition, the growing trend indicates a rising consumer interest in coffees with fruit-infused flavors.

Recent research not only measures the concentration of phenolic compounds along with their in vitro antioxidant capacity but also evaluates the bioaccessibility of these compounds following in vitro digestion [9–11]. Despite their simplicity, in vitro digestion models have proven to be valuable for predicting the outcomes of in vivo digestion [12]. However, the inconsistency in the parameters of various in vitro digestion models have restricted the comparability of findings across studies. To address this issue, INFOGEST developed a standardized static model to simulate digestion in the upper gastrointestinal tract [13, 14]. Although the INFOGEST in vitro digestion model has previously been employed to investigate how digestion of different food items affects the bioaccessibility of phenolic compounds [15], to the best of our knowledge, no previous study has investigated the effect of the addition of extracts of pineapple by-products on the bioaccessibility of coffee phenolics using the in vitro digestion model of INFOGEST.

Previous studies have focused on extraction and characterization of bromelain from pineapple by-products [16, 17], leaving gaps in specific applications of isolated enzymes in food products. Our research aimed to address this gap through incorporation of bromelain in milk coffee. In light of the above, the objective of this study was to assess the extent to which the inclusion of extracts of pineapple by-products, including shell, crown and core, could impact the bioaccessibility of coffee phenolics when combined with milk. Following the measurement of the proteolytic activity of pineapple by-products, the standardized in vitro digestion model of INFOGEST was employed to assess changes in total phenolic content, total antioxidant capacity, and individual phenolic compounds of different coffee formulations.

## Materials and Methods

The materials and methods are presented in the Supplemental Material.

## Results and Discussion

### Proteolytic Activity

The proteolytic activities of pineapple shell, crown and core were found to be 439.9 ± 30.9, 406.3 ± 25.6, and 424.4 ± 29.1 U/mL, respectively, which were statistically not significant from each other (*p* > 0.05). The results obtained are observed to be higher than the data reported in the literature (85.9–199.8 U/mL) [18–20]. These variations could be attributed to the origin or variety of pineapple, as well as the extraction method employed. In this study, bromelain extraction was assisted with ultrasound, aiming to enhance surface contact between the pineapple by-products and solvent. Ultrasound-assisted extraction may enhance cell wall permeability through induction of oscillation, cavitation, microstreaming, and acoustic streaming, thus promoting effective mass transfer [21, 22].

### Total Phenolic Content (TPC)

The changes in the TPC of coffee formulations during in vitro digestion are presented in Table 1. For undigested samples, addition of milk alone (F1) or extracts isolated from different pineapple by-products alone (F5–F7) did not cause a significant difference in the TPC of coffee (F0) (*p* > 0.05). Likewise, the TPC of coffee formulations containing both milk and by-product extracts (F2–F4) were not statistically different from those of the coffee alone (F0) (*p* > 0.05). However, the TPC of F2, containing both milk and pineapple shell extract, was found to be significantly lower than that of F5 (11%), which contained pineapple shell extract alone (*p* < 0.05), suggesting the inhibitory effect of milk on the recovery of phenolics in the presence of pineapple shell extract. After gastric digestion, the outcomes varied, with some formulations showing enhancement, some showing reduction, and others demonstrating no change in TPC compared to undigested formulations. After subsequent intestinal digestion, most samples exhibited higher TPC compared to those observed during gastric digestion and the bioaccessibility values varied between 91 and 129%. The increased TPC following intestinal digestion may be attributed to prolonged extraction time and/or the influence of intestinal digestive enzymes, which promote the release of phenolics [23]. The breakdown of phenolic compounds with a high degree of polymerization or conjugation in their respective derivatives could also contribute to obtained results [24]. It has been observed that the inclusion of extracts from crown or core enhances the bioaccessbility of phenolics both in black and milk coffee (from 93% (F0) to 105–116% (F6, F7) and 114% (F1) to 126–129% (F3, F4), respectively), while this effect was not observed for the shell (F2, F5). The optimal pH for bromelain isolated from pineapple stems is reported to be approximately 7, with its ideal conditions for activity ranging between 50 and 60 °C. In contrast, bromelain derived from the fruity parts exhibits an optimal pH range of 3–8, while its maximum temperature range varies widely from 37 to 70 °C [8]. As the proteolytic activity of extracts from pineapple by-products was measured only at physiological pH, it is possible that the enzyme derived from the shell may not exhibit the intended activity under simulated in vitro digestion conditions. Nevertheless, the TPC assay utilizing the Folin–Ciocalteu reagent lacks specificity for phenolic compounds. The presence of reducing agents such as ascorbic acid, citric acid, simple sugars, or specific amino acids may introduce interference with the analysis, potentially resulting in an overestimation of TPC [25]. Given this limitation, we decided to complement these findings by determining individual phenolic compounds using HPLC-PDA.

**Table 1:** Changes in the total phenolic content and total antioxidant capacity of coffee formulations during in vitro digestion

Sample	Undigested	Gastric digestion	Intestinal digestion	Bioaccessibility
**Total phenolic content (mg GAE/100 mL)**				
*Formulations*				
F0	153.4 ± 6.7 ^AB, a^	80.2 ± 12.2 ^D, b^	142.1 ± 6.5 ^D, a^	93% ^E^
F1	153.5 ± 7.5 ^AB, b^	158.1 ± 11.8 ^B, b^	174.4 ± 15.3 ^BC, a^	114% ^B^
F2	146.5 ± 11.7 ^B, a^	158.3 ± 6.4 ^B, a^	145.1 ± 15.8 ^D, a^	99% ^D^
F3	157.3 ± 10.5 ^AB, c^	185.3 ± 9.6 ^A, b^	203.3 ± 5.1 ^A, a^	129% ^A^
F4	154.4 ± 10.7 ^AB, b^	179.2 ± 9.6 ^A, a^	195.1 ± 19.0 ^AB, a^	126% ^A^
F5	164.4 ± 8.8 ^A, a^	134.5 ± 5.8 ^C, c^	149.8 ± 12.1 ^CD, b^	91% ^E^
F6	156.1 ± 14.5 ^AB, b^	143.6 ± 12.5 ^C, b^	180.4 ± 14.9 ^AB, a^	116% ^B^
F7	160.8 ± 11.6 ^AB, a^	174.5 ± 5.5 ^A, a^	169.1 ± 16.9 ^BCD, a^	105% ^C^
*Controls*				
Shell	6.5 ± 0.7 ^A, c^	25.5 ± 1.8 ^A, b^	43.8 ± 3.9 ^A, a^	
Crown	0.5 ± 0.1 ^C, c^	21.9 ± 1.3 ^B, b^	45.9 ± 3.6 ^A, a^	
Core	2.9 ± 0.3 ^B, c^	24.0 ± 2.0 ^AB, b^	43.0 ± 3.0 ^A, a^	
**Total antioxidant capacity (mg TE/100 mL)**				
**DPPH**				
*Formulations*				
F0	535.2 ± 15.5 ^BC, a^	378.2 ± 19.2 ^A, b^	286.7 ± 35.8 ^CD, c^	54% ^B^
F1	571.2 ± 24.5 ^AB, a^	357.6 ± 10.3 ^BC, b^	321.6 ± 23.3 ^C, c^	56% ^B^
F2	484.1 ± 25.4 ^DE, a^	302.7 ± 9.1 ^E, b^	255.2 ± 25.1 ^D, c^	53% ^B^
F3	499.5 ± 38.9 ^CDE, a^	359.2 ± 7.2 ^BC, c^	434.0 ± 31.1 ^B, b^	87% ^A^
F4	469.0 ± 43.2 ^E, a^	372.1 ± 13.1 ^AB, b^	409.6 ± 34.6 ^B, b^	87% ^A^
F5	483.3 ± 20.3 ^DE, a^	284.2 ± 15.1 ^E, c^	421.0 ± 16.3 ^B, b^	87% ^A^
F6	518.0 ± 23.1 ^CD, a^	346.3 ± 15.4 ^CD, c^	436.8 ± 21.3 ^B, b^	84% ^A^
F7	589.9 ± 33.7 ^A, a^	338.1 ± 7.7 ^D, c^	506.3 ± 27.0 ^A, b^	86% ^A^
*Controls*				
Shell	12.6 ± 1.2 ^A, b^	5.1 ± 0.9 ^B, c^	21.0 ± 1.9 ^AB, a^	
Crown	4.2 ± 0.5 ^C, b^	5.7 ± 0.3 ^B, b^	20.4 ± 2.1 ^B, a^	
Core	8.1 ± 0.5 ^B, b^	7.7 ± 0.7 ^A, b^	23.3 ± 2.7 ^A, a^	
**CUPRAC**				
*Formulations*				
F0	272.9 ± 25.7 ^B, b^	297.3 ± 13.5 ^A, ab^	305.1 ± 19.4 ^AB, a^	112% ^A^
F1	282.6 ± 24.5 ^B, ab^	253.9 ± 24.8 ^C, b^	300.4 ± 27.3 ^AB, a^	106% ^BC^
F2	282.7 ± 14.4 ^B, a^	262.2 ± 10.0 ^BC, b^	247.2 ± 15.1 ^D, b^	87% ^E^
F3	288.5 ± 21.3 ^B, a^	269.6 ± 20.3 ^ABC, a^	291.3 ± 28.5 ^ABC, a^	101% ^CD^
F4	286.9 ± 15.8 ^B, b^	272.3 ± 23.1 ^ABC, b^	319.4 ± 25.9 ^A, a^	111% ^AB^
F5	300.8 ± 28.8 ^B, a^	250.5 ± 20.9 ^C, b^	254.3 ± 15.4 ^CD, b^	85% ^E^
F6	305.0 ± 14.3 ^B, a^	274.1 ± 9.0 ^ABC, b^	302.2 ± 27.4 ^AB, a^	99% ^D^
F7	349.6 ± 22.2 ^A, a^	287.0 ± 25.4 ^AB, b^	274.1 ± 16.7 ^BCD, b^	78% ^F^
*Controls*				
Shell	8.0 ± 0.6 ^A, a^	1.8 ± 0.3 ^A, b^	0.5 ± 0.1 ^B, c^	
Crown	0.3 ± 0.1 ^C, a^	0.4 ± 0.1 ^C, a^	nd	
Core	1.8 ± 0.6 ^B, a^	1.2 ± 0.2 ^B, a^	1.0 ± 0.5 ^A, a^	

The values provided in this table represent the mean values ± standard deviation derived from three biological replicates (repetitions with the same formulation) and three technical replicates (measurements per assay). Statistically significant differences, which are assigned separately among the beverages and control groups, are denoted by uppercase letters in the columns and lowercase letters in the rows (*p* < 0.05). Abbreviations used for formulations are expressed in the supplementary material Table S1

### Total Antioxidant Capacity (TAC)

The changes in the TAC of coffee formulations during in vitro digestion are measured using CUPRAC and DPPH assays, and the results are presented in Table 1. Among the undigested samples, the coffee formulation containing core extract (F7) was found to possess the highest antioxidant capacity in both assays. For the CUPRAC assay results, no statistically significant differences were observed in the TAC among all other formulations (F0–F6) (*p* > 0.05). However, DPPH assay results showed that addition of pineapple by-product extracts to both the black and milk coffee results in reduced TAC (3–18%) in undigested samples except for F7. Following gastric digestion, the outcomes varied; however, for the majority of the samples, there was a reduction in TAC compared to undigested samples. Subsequent intestinal digestion, on the other hand, resulted in an enhanced TAC. The increase in antioxidant potential observed during the intestinal phase may be attributed to the generation of new oxidation products, exhibiting greater antioxidant activity than their precursors. Additionally, it could also result from the presence of other antioxidant molecules that are not phenolic compounds [15]. The bioaccessibility values varied between 53 and 87% and 78–112% according to DPPH and CUPRAC assays, respectively. In line with the TPC results, the inclusion of extracts from both the crown and core in black and milk coffee led to increased bioaccessibility of antioxidants, as indicated by the DPPH assay (from 54% (F0) to 84–86% (F6, F7) and 56% (F1) to 87% (F3, F4), respectively). Conversely, the CUPRAC assay results generally indicated that the addition of pineapple by-product extracts to black or milk coffee had a negative effect on the bioaccessibility of antioxidants. Both the DPPH and CUPRAC assays offer the advantages of simplicity and cost-effectiveness, requiring no specialized equipment. On the other hand, DPPH assay is only suitable for lipophilic antioxidants, whereas CUPRAC assay can measure both hydrophilic and lipophilic antioxidants. Some additional advantages of the CUPRAC assay include the fact that it is conducted at a pH level close to physiological pH. Furthermore, factors such as light, air, humidity, and pH within reasonable limits do not significantly affect the reaction. Additionally, it is more reproducible compared to other ET-based assays [25]. Nevertheless, relying on a single assay to determine the TAC of food products is not entirely reliable. Thus, conducting multiple TAC assays with different mechanisms is essential to ensure more accurate and dependable results, as demonstrated in this study using the ET-based CUPRAC and mixed-mode DPPH assays.

### Individual Phenolic Compounds

In all coffee formulations, four major phenolic compounds were detected: chlorogenic acid (5-*O*-caffeoylquinic acid or 5-CQA), neochlorogenic acid (3-CQA), cryptochlorogenic acid (4-CQA) (Table 2), and gallic acid (Table 3). Consistent with previous reports [26, 27], chlorogenic acid was identified as the predominant compound in all coffee formulations, representing 75–77% of total CQAs. In addition, shell and crown extracts were found to contain gallic and ferulic acids (Table 3), which is also in agreement with previous findings reported in the literature [28, 29]. For undigested samples, the addition of milk alone (F1) or extracts isolated from different pineapple by-products alone (F5–F7), or their combinations (F2–F4), generally did not significantly alter the total caffeoylquinic acid and gallic acid contents compared to black coffee (F0) (*p* > 0.05). Gastric digestion led to significant reductions in the total caffeoylquinic acid content (*p* < 0.05), which further decreased during subsequent intestinal digestion. In fact, neochlorogenic and cryptochlorogenic acids were not detected after intestinal digestion. These substantial losses observed may result from the instability of CQAs in aqueous solutions. Additionally, in vitro digestion could induce the isomerization of CQAs and/or the formation of additional degradation products [30]. However, a different trend was observed for gallic acid during in vitro digestion. While gallic acid levels reduced following gastric digestion (*p* < 0.05), increases were noted after intestinal digestion. This observation may be related to the release of gallic acid from pineapple by-product extracts during intestinal digestion. When examining the bioaccessibility values, it becomes apparent that they are notably low for CQAs (< 1%), while for gallic acid they ranged between 52 and 155%. The incorporation of core extracts enhanced the bioaccessibility of CQAs and gallic acid in milk coffee (from 0.72% (F1) to 0.85% (F4) and 109% (F1) to 155% (F4), respectively), confirming our hypothesis regarding the favorable effect of bromelain on the recovery of phenolic compounds. On the other hand, this effect was not evident in coffee formulations containing extracts from the shell and crown. It is possible that shell and crown extracts also contain fiber, which could potentially hinder the bioaccessibility of phenolic compounds. Fiber-bound compounds tend to be poorly extracted and have limited solubility in gastrointestinal fluids [31].

**Table 2:** Changes in the caffeoylquinic acids (CQA) of coffee formulations during in vitro digestion (mg/100 mL)

Sample	Undigested	Gastric digestion	Intestinal digestion	Bioaccessibility
**Neochlorogenic acid (3-CQA)**				
Formulations				
F0	9.77 ± 0.20 ^A, a^	2.55 ± 0.03 ^A, b^	nd	n/a
F1	9.35 ± 0.06 ^A, a^	2.48 ± 0.09 ^A, b^	nd	n/a
F2	8.85 ± 0.69 ^A, a^	2.24 ± 0.14 ^AB, b^	nd	n/a
F3	9.10 ± 0.12 ^A, a^	2.60 ± 0.13 ^A, b^	nd	n/a
F4	8.32 ± 1.39 ^A, a^	2.51 ± 0.01 ^A, b^	nd	n/a
F5	9.43 ± 0.09 ^A, a^	1.70 ± 0.05 ^B, b^	nd	n/a
F6	9.31 ± 0.14 ^A, a^	2.50 ± 0.17 ^A, b^	nd	n/a
F7	9.64 ± 0.01 ^A, a^	1.69 ± 0.34 ^B, b^	nd	n/a
*Controls*				
Shell	nd	nd	nd	
Crown	nd	nd	nd	
Core	nd	nd	nd	
**Chlorogenic acid (5-CQA)**				
Formulations				
F0	35.10 ± 2.54 ^AB, a^	4.76 ± 0.13 ^A, b^	0.32 ± 0.06 ^A, b^	0.92% ^B^
F1	32.29 ± 3.15 ^AB, a^	4.70 ± 0.28 ^A, b^	0.30 ± 0.09 ^A, b^	0.94% ^B^
F2	31.56 ± 3.86 ^AB, a^	6.91 ± 0.63 ^A, b^	0.16 ± 0.02 ^A, b^	0.49% ^F^
F3	30.98 ± 0.34 ^AB, a^	4.81 ± 0.32 ^A, b^	0.21 ± 0.03 ^A, c^	0.68% ^D^
F4	28.45 ± 3.91 ^B, a^	4.63 ± 0.02 ^A, b^	0.32 ± 0.01 ^A, b^	1.13% ^A^
F5	34.79 ± 0.31 ^AB, a^	5.81 ± 0.09 ^A, b^	0.21 ± 0.03 ^A, c^	0.60% ^E^
F6	34.39 ± 0.80 ^AB, a^	6.16 ± 1.60 ^A, b^	0.27 ± 0.06 ^A, c^	0.78% ^C^
F7	38.61 ± 0.33 ^A, a^	4.64 ± 0.35 ^A, b^	0.18 ± 0.01 ^A, c^	0.46% ^F^
Controls				
Shell	nd	nd	nd	
Crown	nd	nd	nd	
Core	nd	nd	nd	
**Cryptochlorogenic acid (4-CQA)**				
Formulations				
F0	1.50 ± 0.04 ^AB^	nd	nd	n/a
F1	0.65 ± 0.04 ^C^	nd	nd	n/a
F2	0.77 ± 0.12 ^BC^	nd	nd	n/a
F3	1.18 ± 0.35 ^ABC^	nd	nd	n/a
F4	0.90 ± 0.24 ^BC^	nd	nd	n/a
F5	1.85 ± 0.16 ^A^	nd	nd	n/a
F6	1.74 ± 0.18 ^A^	nd	nd	n/a
F7	1.83 ± 0.30 ^A^	nd	nd	n/a
Controls				
Shell	nd	nd	nd	
Crown	nd	nd	nd	
Core	nd	nd	nd	
**Total caffeoylquinic acids**				
Formulations				
F0	46.38 ± 2.69 ^AB, a^	7.31 ± 0.16 ^AB, b^	0.32 ± 0.06 ^A, c^	0.69% ^B^
F1	42.29 ± 3.25 ^AB, a^	7.18 ± 0.37 ^AB, b^	0.30 ± 0.09 ^A, b^	0.72% ^B^
F2	41.18 ± 4.67 ^AB, a^	9.14 ± 0.77 ^A, b^	0.16 ± 0.02 ^A, b^	0.38% ^F^
F3	41.26 ± 0.80 ^AB, a^	7.40 ± 0.45 ^AB, b^	0.21 ± 0.03 ^A, c^	0.51% ^D^
F4	37.67 ± 5.55 ^B, a^	7.15 ± 0.04 ^AB, b^	0.32 ± 0.01 ^A, b^	0.85% ^A^
F5	46.08 ± 0.56 ^AB, a^	7.51 ± 0.14 ^AB, b^	0.21 ± 0.03 ^A, c^	0.45% ^E^
F6	45.45 ± 0.76 ^AB, a^	8.66 ± 1.43 ^AB, b^	0.27 ± 0.06 ^A, c^	0.59% ^C^
F7	50.08 ± 0.64 ^A, a^	6.33 ± 0.01 ^B, b^	0.18 ± 0.01 ^A, c^	0.36% ^F^
Controls				
Shell	nd	nd	nd	
Crown	nd	nd	nd	
Core	nd	nd	nd	

The values provided in this table represent the mean values ± standard deviation derived from three biological replicates (repetitions with the same formulation) and three technical replicates (measurements per assay). Neochlorogenic acid and cryptochlorogenic acid are quantified relative to chlorogenic acid. Statistically significant differences, which are assigned separately among the beverages and control groups, are denoted by uppercase letters in the columns and lowercase letters in the rows (*p* < 0.05). nd: not detected; n/a: not applicable. Abbreviations used for formulations are expressed in the supplementary material Table S1

**Table 3:** Changes in the gallic and ferulic acids of coffee formulations during in vitro digestion (mg/100 mL)

Sample	Undigested	Gastric digestion	Intestinal digestion	Bioaccessibility
**Gallic acid**				
Formulations				
F0	2.32 ± 0.28 ^AB, a^	0.048 ± 0.004 ^A, b^	1.79 ± 0.90 ^A, a^	77% ^D^
F1	1.97 ± 0.01 ^ABC, a^	0.026 ± 0.002 ^BC, b^	2.16 ± 0.37 ^A, b^	109% ^B^
F2	1.49 ± 0.13 ^BC, a^	0.021 ± 0.001 ^C, b^	1.54 ± 0.03 ^A, b^	103% ^C^
F3	1.51 ± 0.01 ^BC, a^	0.025 ± 0.003 ^C, b^	1.70 ± 0.12 ^A, b^	113% ^B^
F4	1.35 ± 0.39 ^C, a^	0.024 ± 0.003 ^C, b^	2.09 ± 0.04 ^A, b^	155% ^A^
F5	2.46 ± 0.01 ^A, a^	0.035 ± 0.001 ^B, c^	1.28 ± 0.25 ^A, b^	52% ^F^
F6	2.29 ± 0.36 ^AB, a^	0.050 ± 0.003 ^A, b^	1.46 ± 0.01 ^A, b^	64% ^E^
F7	2.55 ± 0.04 ^A, a^	0.047 ± 0.001 ^A, c^	1.39 ± 0.02 ^A, b^	54% ^F^
Controls				
Shell	0.15 ± 0.01 ^A, b^	0.29 ± 0.06 ^AB, b^	1.28 ± 0.29 ^A, a^	
Crown	0.08 ± 0.01 ^B, b^	0.10 ± 0.01 ^B, b^	1.59 ± 0.05 ^A, a^	
Core	0.07 ± 0.02 ^B, b^	0.29 ± 0.05 ^A, b^	1.56 ± 0.09 ^A, a^	
**Ferulic acid**				
Formulations				
F0	nd	nd	nd	n/a
F1	nd	nd	nd	n/a
F2	nd	nd	nd	n/a
F3	nd	nd	nd	n/a
F4	nd	nd	nd	n/a
F5	nd	nd	nd	n/a
F6	nd	nd	nd	n/a
F7	nd	nd	nd	n/a
Controls				
Shell	0.39 ± 0.03 ^A, a^	0.137 ± 0.001 ^b^	nd	
Crown	0.13 ± 0.01 ^B^	nd	nd	
Core	nd	nd	nd	

The values provided in this table represent the mean values ± standard deviation derived from three biological replicates (repetitions with the same formulation) and three technical replicates (measurements per assay). Statistically significant differences, which are assigned separately among the beverages and control groups, are denoted by uppercase letters in the columns and lowercase letters in the rows (*p* < 0.05). nd: not detected; n/a: not applicable. Abbreviations used for formulations are expressed in the supplementary material Table S1

## Conclusions

To the best of our knowledge, no previous study has investigated the effect of the addition of pineapple by-products on the bioaccessibility of coffee phenolics using the in vitro digestion model of INFOGEST. The results revealed that incorporating extracts from the crown or core in both black and milk coffee increases the bioaccessibility of total phenolics and antioxidants. Additionally, adding core extracts also enhances the bioaccessibility of CQAs and gallic acid in milk coffee. Overall, we demonstrated that by-products of pineapple, particularly the crown and core, which are discarded as waste, have a potential to be utilized in milk coffee. However, the findings of this study are limited to the specific formulations examined. Other factors such as the proportions of coffee, milk, and pineapple by-products, as well as the temperature during mixing, could influence the bioaccessibility of phenolic compounds and need further investigation in future research. Moreover, future investigations should also conduct research on toxicological data before these materials can be utilized as food ingredients. Finally, although often ignored, the consumer acceptance of food products incorporating waste materials also should be examined.

### Electronic Supplementary Material

Below is the link to the electronic supplementary material.

Supplementary Material 1

## Data Availability

The authors declare that the data supporting the findings of this study are available within the paper and its Supplemental Material. Should any raw data files be needed they are available from the corresponding author upon reasonable request.
