# Supplementary material for: Valorization of Pineapple (Ananas comosus) By-Products in Milk Coffee Beverage: Influence on Bioaccessibility of Phenolic Compounds
Source: Plant Foods Hum Nutr. 2024 May 2;79(2):300–7. doi: 10.1007/s11130-024-01183-w (PMC11178570; doi:10.1007/s11130-024-01183-w)
Supplement: Supplementary file 1 — Supplementary Material 1 [file 11130_2024_1183_MOESM1_ESM.docx]

**Materials and methods**

**Chemicals**

All chemicals and reagents utilized in this study including potassium phosphate monobasic (≥99.0%), potassium phosphate dibasic (≥99.0%), casein from bovine milk (87-94% protein basis), tricholoroacetic acid (≥98%), sodium carbonate (≥99.5%), Folin-Ciocalteu′s phenol reagent (pH<0.5), L-tyrosine (≥98%), potassium chloride (≥99.0%), sodium bicarbonate (≥99.5%), sodium chloride (≥99.0%), magnesium chloride hexahydrate (≥99.0%), ammonium carbonate (≥30.0% NH_3_ basis), calcium chloride (≥97%), pepsin from porcine gastric mucosa (≥2,500 units/mg protein), pancreatin from porcine pancreas (8 × USP), bile extract porcine, hydrochloric acid (37%), sodium hydroxide (97%), gallic acid (≥98.0%), 2,2-diphenyl-1-picrylhydrazyl (DPPH), methanol (≥99.8%), (±)-6-Hydroxy-2,5,7,8-tetramethylchromane-2-carboxylic acid (Trolox^®^, 97%), copper (II) chloride (97%), neocuproine (≥98%), ammonium acetate (≥98%), acetonitrile (HPLC grade, ≥99.9%), trifluoroacetic acid (HPLC grade, ≥99.0%), chlorogenic acid (HPLC grade) and ferulic acid (HPLC grade) were obtained from Sigma–Aldrich (Steinheim, Germany).

**Extraction of Bromelain**

Pineapples imported from Monaco/Monte Carlo were purchased from a local market in Istanbul, Türkiye. Shell, crown and core were removed from the fruits. For each by-product, 80 g of sample was homogenized with distilled water at 10°C. Subsequently, each sample underwent a 30 min ultrasonic bath (VWR USC900TH; Radnor, PA, USA) treatment, followed by filtration through a cloth. The mixtures were transferred to falcon tubes, centrifuged (Hettich Universal 32R; Tuttlingen, Germany) at 5000 rpm for 12 min, and then stored at -20°C until analysis [1].

**Determination of Proteolytic Activity**

Proteolytic activity of pineapple by-products was determined as described previously [2]. Briefly, 1 mL of enzyme solution, the supernatant of the previous section, was combined with 5 mL of 0.05 M potassium phosphate buffer containing 0.65% (w/v) casein at pH 7.5. The mixture was gently shaken in a water bath (Memmert SV 1422; Nürnberg, Germany) at 37°C for 10 min. To stop the reaction, 5 mL of 110 mM trichloroacetic acid was introduced, and subsequently, the mixtures were filtered through 0.45 μm member filters. The supernatant was combined with 5 mL of 500 mM sodium carbonate solution, followed by the addition of 1 mL of 20% (v/v) Folin Ciocalteu's phenol reagent. The mixture was then incubated in a water bath at 37°C for 30 min. Afterwards, the absorbance was measured at 660 nm using a UV–Vis spectrophotometer (Optima SP-3000 nano, Tokyo, Japan). L-tyrosine was used as a reference standard for the calibration curve (𝑦=0.0029𝑥−0.0084; *R^2^*=0.998), and bromelain activity was expressed as U/mL, equivalent to μm L-tyrosine released within the concentration range of 0.05-0.5 mM. All extracts were measured in five replicates.

**Coffee Formulations and *In Vitro* Digestion**

1 ± 0.01 g of instant gold coffee was mixed with ingredients listed in Table S1. Eight different coffee formulations, along with pineapple by-product extracts alone, were subjected to the *in vitro* digestion model of INFOGEST in triplicate, as described previously [3, 4]. The salivary, gastric, and intestinal fluids were prepared in accordance with the specifications outlined in the protocol. To simulate oral digestion, 5 mL of coffee formulations were combined with 3.5 mL of salivary fluid and 25 μL of calcium chloride. The volume was then adjusted to 10 mL using distilled water, and the mixture was incubated in a shaking water bath at 37°C for 2 min. Afterwards, to simulate gastric digestion, 7.5 mL of gastric fluid, 1.6 mL of pepsin from porcine gastric mucosa (25,000 U/mL) and 5 μL of calcium chloride were added to the mixture. The pH was lowered to 3 using 1 M HCl, and the volume was adjusted to 20 mL with distilled water. The mixtures were placed in a water bath and shaken at 37°C for 2 h, and after gastric digestion was concluded, 5 mL samples were collected. Following that, to simulate intestinal digestion, 8.25 mL of intestinal fluid, 3.75 mL of pancreatin from porcine pancreas (800 U/mL), 1.875 mL of 160 mM bile from porcine and 30 μL of calcium chloride were added to the remaining mixture. The pH was increased to 7 using 1 M NaOH, and the volume was adjusted to 30 mL with distilled water. The mixtures were again placed in a water bath and shaken at 37°C for 2 h, and after the conclusion of intestinal digestion, samples were collected. Simultaneously, a blank sample without the addition of coffee formulations was incubated under the same conditions. The blank was employed to correct any interferences arising from the digestive fluids. All collected samples were centrifuged at 5000 rpm for 10 min, and stored at -20°C until analysis. The bioaccessibility was calculated using the following formula:

$$\mathrm{Bioaccessibility}\left( \% \right)=\frac{Phenolics/Antioxidants after intestinal digestion}{Phenolics/Antioxidants in undigested extract}\times100$$

**Determination of Total Phenolic Content**

Total phenolic content was determined using Folin–Ciocalteu reagent as described previously [5]. Fifteen microliters of the extracts were inserted into 96-well plates, followed by the addition of 112.5 μL of Folin-Ciocalteu reagent. The mixture was then allowed to stand at room temperature for 5 min. Subsequently, 112.5 μL of sodium carbonate solution was introduced, and the mixture was incubated in the dark at room temperature for 1 h. Afterwards, the absorbance was measured at 765 nm using a using a microplate reader (BioTek Synergy HT; Winooski, VT, USA). Gallic acid was used as a reference standard for the calibration curve (𝑦=5.3863𝑥+0.0749; *R^2^*=0.998; linear range: 0.01–0.4 mM), and the results were expressed as mg gallic acid equivalent (GAE)/100 mL sample. All extracts were measured in triplicates.

**Determination of Total Antioxidant Capacity**

Total antioxidant capacity was determined with DPPH (2,2-Diphenyl-1-picrylhydrazyl) [6] and CUPRAC (Cupric Ion Reducing Antioxidant Capacity) [7] assays.

For DPPH assay, 10 μL of extracts were mixed with 200 μL of 0.1 mM DPPH reagent dissolved in methanol. The mixture was shaken gently for 10 s and then incubated in the dark at room temperature for 30 min. Afterwards, the absorbance was measured at 517 nm using a microplate reader. Trolox^®^ was used as a reference standard for the calibration curve (𝑦=1.6045𝑥−0.0317; *R^2^*=0.995; linear range: 0.01–0.4 mM), and the results were expressed as mg Trolox^®^ equivalent (TE)/100 mL sample. All extracts were measured in triplicates.

For CUPRAC assay, 7 μL of extracts were sequentially combined with 70 μL of 10 mM copper (II) chloride, 70 μL of 7.5 mM neocuproine, 70 μL of 1 M ammonium acetate and 70 μL of distilled water. The mixture was shaken gently for 10 s and then incubated in the dark at room temperature for 30 min. Afterwards, the absorbance was measured at 450 nm using a microplate reader. Trolox^®^ was used as a reference standard for the calibration curve (𝑦=2.8424𝑥+0.0691; *R^2^*=0.995; linear range: 0.01–0.4 mM), and the results were expressed as mg Trolox^®^ equivalent (TE)/100 mL sample. All extracts were measured in triplicates.

**Determination of Individual Phenolic Compounds using HPLC-PDA**

The identification and quantification of individual phenolic compounds were conducted using Waters 2695 HPLC coupled to Waters 2996 PDA detector (Waters Co.; Milford, MA, USA) as described previously [8]. Chromatographic separation was achieved with a C18 column (250 × 4.6 mm, 5 μm; Supelcosil, Sigma-Aldrich) at 40°C. Sample injections, consisting of 10 μL extracts filtered through 0.45 μm membrane filters, were introduced into the system using mobile phase A (TFA:MilliQ water, 1:1000, v/v) and mobile phase B (TFA:acetonitrile, 1:1000, v/v) at a flow rate of 1 mL/min. The linear gradient proceeded as follows: 5% B at 0 min, 35% B at 0–45 min, 75% B at 45–47 min, 35% B at 47–49 min, returning to initial conditions at 50 min. Spectral measurements were taken at 280 and 312 nm. For the quantification of phenolic compounds chlorogenic acid, gallic acid and ferulic acid standards were used. The phenolic standards exhibited good linearity (*R^2^*≥0.99) within the established range (0.1–200 ppm). The results were expressed as mg/100 mL sample. Representative chromatograms are shown in Figure S1.

**Statistical Analysis**

All experiments were carried out with minimum three biological replicates (repetitions with the same formulation) and three technical replicates (measurements per assay). The obtained data underwent statistical analysis utilizing SPSS software (version 28; Chicago, IL, USA). Treatment comparisons were conducted through one-way ANOVA, followed by a Tukey post hoc test, with a significance level set at *p* < 0.05.

**References**

1. Mohan R, Sivakumar V, Rangasamy T, Muralidharan C (2016) Optimisation of bromelain enzyme extraction from pineapple (Ananas comosus) and application in process industry. Am J Biochem Biotechnol 12:188–195

2. Mala T, Sadiq MB, Anal AK (2021) Comparative extraction of bromelain and bioactive peptides from pineapple byproducts by ultrasonic‐ and microwave‐assisted extractions. J Food Process Eng 44:e13709. https://doi.org/10.1111/jfpe.13709

3. Minekus M, Alminger M, Alvito P, et al (2014) A standardised static *in vitro* digestion method suitable for food – an international consensus. Food Funct 5:1113–1124. https://doi.org/10.1039/C3FO60702J

4. Brodkorb A, Egger L, Alminger M, et al (2019) INFOGEST static in vitro simulation of gastrointestinal food digestion. Nat Protoc 14:. https://doi.org/10.1038/s41596-018-0119-1

5. Spanos GA, Wrolstad RE (1990) Influence of processing and storage on the phenolic composition of Thompson Seedless grape juice. J Agric Food Chem 38:1565–1571. https://doi.org/10.1021/jf00097a030

6. Kumaran A, Karunakaran RJ (2006) Antioxidant and free radical scavenging activity of an aqueous extract of Coleus aromaticus. Food Chem 97:109–114. https://doi.org/10.1016/J.FOODCHEM.2005.03.032

7. Apak R, Güçlü K, Özyürek M, Karademir SE (2004) Novel Total Antioxidant Capacity Index for Dietary Polyphenols and Vitamins C and E, Using Their Cupric Ion Reducing Capability in the Presence of Neocuproine:  CUPRAC Method. J Agric Food Chem 52:7970–7981. https://doi.org/10.1021/jf048741x

8. Kamiloglu S, Ozdal T, Bakir S, Capanoglu E (2022) Bioaccessibility of terebinth (Pistacia terebinthus L.) coffee polyphenols: Influence of milk, sugar and sweetener addition. Food Chem 374:131728. https://doi.org/10.1016/j.foodchem.2021.131728

**Table S1.** Coffee beverage formulations

| **Formulation (F)** | **Coffee** | **Water (92°C)** | **Skim milk**^1^ | **Bromelain extract (10°C)** | | |
| --- | --- | --- | --- | --- | --- | --- |
|  |  |  |  | **Shell** | **Crown** | **Core** |
| F0 | 1 g | 40 mL | - | - | - | - |
| F1 | 1 g | 30 mL | 10 mL | - | - | - |
| F2 | 1 g | 20 mL | 10 mL | 10 mL | - | - |
| F3 | 1 g | 20 mL | 10 mL | - | 10 mL | - |
| F4 | 1 g | 20 mL | 10 mL | - | - | 10 mL |
| F5 | 1 g | 30 mL | - | 10 mL | - | - |
| F6 | 1 g | 30 mL | - | - | 10 mL | - |
| F7 | 1 g | 30 mL | - | - | - | 10 mL |

^1^ Contains 0.1% fat.

**4-CQA**

**5-CQA**

**GA**

**3-CQA**

(a)

**GA**

**4-CQA**

**5-CQA**

**3-CQA**

(b)

**GA**

**4-CQA**

**5-CQA**

**3-CQA**

(c)

**GA**

**4-CQA**

**5-CQA**

**3-CQA**

(d)

**GA**

**4-CQA**

**5-CQA**

**3-CQA**

(e)

**GA**

**4-CQA**

**5-CQA**

**3-CQA**

(f)

**GA**

**4-CQA**

**5-CQA**

**3-CQA**

(g)

**GA**

**4-CQA**

**5-CQA**

**3-CQA**

(h)

**FA**

**GAv**

(i)

**FA**

**GAv**

(j)

**GAv**

(k)

**Supplementary Fig. 1:** HPLC chromatograms at 312 nm for (a) F0, (b) F1, (c) F2, (d) F3, (e) F4, (f) F5, (g) F6, (h) F7 as well as at 280 nm for (i) shell, (j) crown and (k) core samples before digestion (GA: gallic acid; FA: ferulic acid; 3-CQA: Neochlorogenic acid; 5-CQA: Chlorogenic acid; 4-CQA: Cryptochlorogenic acid)
